# Familial confounding of internalising symptoms and obesity in adolescents and young adults; a co-twin analysis

**DOI:** 10.1038/s41366-024-01491-w

**Published:** 2024-02-15

**Authors:** Alexander Charles Campbell, Lucas Calais-Ferreira, Elisabeth Hahn, Frank M. Spinath, John L. Hopper, Jesse T. Young

**Affiliations:** 1 Centre for Epidemiology and Biostatistics, Melbourne School of Population and Global Health, The University of Melbourne, Parkville, VIC Australia; 2 Centre for Adolescent Health, Murdoch Children’s Research Institute, Parkville, VIC Australia; 3 Justice Health Group, School of Population Health, Curtin University, Perth, WA Australia; 4 Centre for Mental Health and Community Wellbeing, Melbourne School of Population and Global Health, The University of Melbourne, Parkville, VIC Australia; 5 Department of Psychology, Saarland University, Saarbruecken, Germany; 6 School of Population and Global Health, The University of Western Australia, Perth, WA Australia; 7 National Drug Research Institute, Curtin University, Perth, WA Australia; 8 Institute for Mental Health Policy Research, Centre for Addiction and Mental Health, Toronto, OC Canada; 9 Dalla Lana School of Public Health, University of Toronto, Toronto, OC Canada

**Keywords:** Risk factors, Endocrine system and metabolic diseases

## Abstract

**Background:**

Obesity and internalising disorders, including depression and anxiety, often co-occur. There is evidence that familial confounding contributes to the co-occurrence of internalising disorders and obesity in adults. However, its impact on this association among young people is unclear. Our study investigated the extent to which familial factors confound the association between internalising disorders and obesity in adolescents and young adults.

**Subjects/methods:**

We used a matched co-twin design to investigate the impact of confounding by familial factors on associations between internalising symptoms and obesity in a sample of 4018 twins aged 16 to 27 years.

**Results:**

High levels of internalising symptoms compared to low levels increased the odds of obesity for the whole cohort (adjusted odds ratio [AOR] = 3.1, 95% confidence interval [CI]: 1.5, 6.8), and in females (AOR = 4.1, 95% CI 1.5, 11.1), but not in males (AOR = 2.8 95% CI 0.8, 10.0). We found evidence that internalising symptoms were associated with an increased between-pair odds of obesity (AOR 6.2, 95% CI 1.7, 22.8), using the paired analysis but not using a within-pair association, which controls for familial confounding. Sex-stratified analyses indicated high internalising symptoms were associated with increased between-pair odds of obesity for females (AOR 12.9, 95% CI 2.2, 76.8), but this attenuated to the null using within-pair analysis. We found no evidence of between or within-pair associations for males and weak evidence that sex modified the association between internalising symptoms and obesity (likelihood ratio test *p* = 0.051).

**Conclusions:**

Some familial factors shared by twins confound the association between internalising symptoms and obesity in adolescent and young adult females. Internalising symptoms and obesity were not associated for adolescent and young adult males. Therefore, prevention and treatment efforts should especially address familial shared determinants of obesity, particularly targeted at female adolescents and young adults with internalising symptoms and those with a family history of these disorders.

## Introduction

Obesity is a chronic disease characterised by excessive accumulation of body fat [1]. The global prevalence of obesity in adolescents and young adults has increased more than five-fold in the last 40 years [2], and 10–15% of adolescents in most high income countries are now affected by obesity [2]. Experiencing obesity is associated with poor health outcomes, including type 2 diabetes, some types of cancers, cardiovascular disease, and significant social stigma which in turn can cause a variety of negative life outcomes [3–5].

Internalising disorders are a broad group of mental illnesses characterised by symptoms of anxiety, somatisation, and depression [6]. In 2013, the global point prevalence of anxiety and depression were estimated at 7.3 and 4.7%, respectively [7, 8].

Obesity and internalising disorders often co-occur. A meta-analysis found individuals with a depressive disorder have a 55% greater risk of developing obesity and individuals with obesity have a 58% greater risk of developing a depressive disorder [9]. Likewise, adolescents with an anxiety disorder are at 32% higher risk of experiencing obesity than adolescents without an anxiety disorder [10].

Adolescence and young adulthood are critical periods for the onset of internalising disorders and obesity; 75% of all individuals who develop an anxiety disorder in their lifetime will experience their first episode by the age of 21 and 50% of all individuals who develop a depressive disorder in their lifetime will experience their first episode by the age of 30 years [11]. Similarly, the largest increase in the prevalence of obesity occurs in adolescence and young adulthood between the ages of 15 and 25 years [12]. Compared to adult-onset obesity, obesity in adolescence is associated with poorer health, including a greater risk of experiencing obesity throughout the life course, experiencing more extreme obesity in adulthood, and faster onset of obesity related diseases [13]. For example, a study of 8834 adolescents aged 12 to 21 years in the United States found that 70% of adolescents with obesity experienced severe obesity as adults (body mass index [BMI] > 40 kg/m^2^) compared to 8% of adolescents who were not obese [14]. A study of 1,462,362 Israeli adolescents found adolescents with obesity were at 13 times higher odds of type 2 diabetes than adolescents who were not obese [15].

The mechanisms through which obesity and internalising disorders influence one another are poorly understood [16, 17]. However, the association between internalising disorders and obesity has been found to be stronger in females than in males [18]. A meta-analysis of longitudinal studies found depression was associated with a 2.5 (95% Confidence interval [CI]: 2.27, 2.91) times higher odds of obesity in adolescent females compared to females without depression and there was no association between depression and obesity in adolescent males (odds ratio [OR] = 1.23, 95% CI: 0.74, 2.04) [19]. Another meta-analysis found women with obesity had 1.37 (95% CI: 1.18, 1.60) times higher odds of an anxiety disorder and men with obesity had 1.26 (95% CI: 1.15, 1.38) times higher odds of an anxiety disorder [10].

Internalising disorders and obesity tend to aggregate within families. Individuals with a first degree relative with depression or anxiety have from 1.1 to 2.0 times greater odds of developing an internalising disorder themselves and have 1.2 to 6.5 times greater odds of developing obesity [20–22]. Familial aggregation occurs because families share environmental and genetic variants that contribute to internalising disorders and obesity at the population level [23]. Studies have found that both genetic variants and environment likely determine this association to some extent, but evidence on their relative contribution is equivocal [24–27]. Specific environmental factors shared by families that increase the odds of internalising disorders and obesity include parental modelling of dietary intake and physical activity, parental mental health, and family socioeconomic position [28–33]. There is also evidence of genetic variants affecting individual differences in internalising disorders and obesity from both molecular and twin studies [34, 35].

Most causal inference in this area has been derived from typical cohort studies which either implicitly assume the influence of familial aggregation on the association between internalising disorders and obesity is not important or ignore this possibility altogether. As a result, it is possible that the widely-reported association between internalising disorders and obesity may be confounded by genetic and environmental risks that are shared by family members. If so, interventions in this area, targeted at individual behaviour change alone, are unlikely to be effective.

Twin cohorts represent a powerful ‘natural experiment’ that provides an opportunity to investigate if genetic and environmental factors shared by families confound the association between internalising disorders and obesity [36]. Matched co-twin control designs allow the use of twin pairs which differ in the outcome of interest as a matched case-control pair. As twins share approximately 50 or 100% of genetic variants (in dizygotic and monozygotic twins, respectively) and many early life environmental exposures, treating twin pairs as matched case-control pairs implicitly adjusts for unmeasured shared genetic and environmental risks. This allows for estimates of the association between internalising disorders and obesity that control for familial confounding [36].

To our knowledge, only two previous studies have employed matched co-twin designs to evaluate the effect of familial confounding on the association between internalising disorders and obesity. In a study of 2831 adult United States twins (mean age 35 years) depression was associated with higher BMI in adult men and women, while anxiety was only associated with higher BMI in women [37]. After controlling for familial confounding, there was no evidence of associations between anxiety or depression and BMI in adult men or women. Similarly, in a combined sample of 8224 Finnish and 1105 United States adult twins (mean age 44 years) it was found that adjusting for confounding by genetic and environmental factors shared by twins attenuated the association between depressive symptoms and obesity to the null [35].

Both studies included predominantly middle-aged adults and did not include adolescents aged under 18 years. Consequently, there is little evidence on the effects of familial confounding on the association of internalising disorders and obesity at the developmental periods when these disorders tend to onset. Furthermore, only one of these studies stratified analyses by sex, despite there being evidence that sex modifies the association between internalising symptoms and obesity [18, 38]. As a result, little is known about how familial confounding may impact the relationship between internalising symptoms and obesity in adolescents and young adults and how these effects may differ by sex. By applying a matched co-twin design in a large cohort of adolescent and young adult twins in Germany, we aimed to: (1) examine the relationship between internalising symptoms and obesity while adjusting for familial confounding; and (2) investigate whether sex modifies the individual and within-pair associations between internalising symptoms and obesity.

## Subjects and Methods

### Subjects

**Fig. 1:**
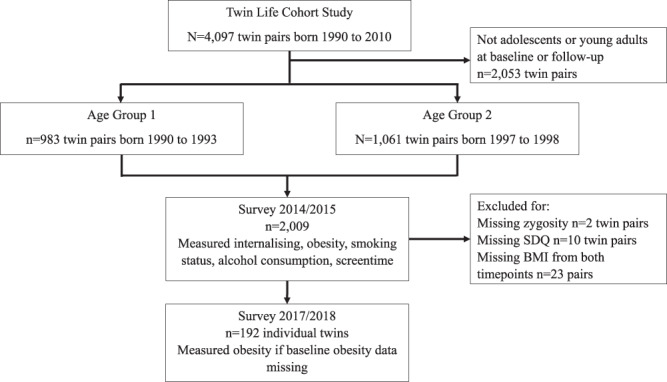
Flow chart depicting the selection of participants for this study from the Twin Life Cohort Study. Arrows pointing the right indicate participants who were excluded from the final sample.

The German Twin Family Panel (Twin Life) study is a longitudinal twin-family study of 4097 same-sex twin pairs living in Germany [39]. We used data on 2044 twin pairs from the two oldest age groups of the Twin Life study, born in 1990 to 1993 and 1997 to 1998, as these were the only age groups where all participants were adolescents (aged 16–24 years) at baseline or young adults (aged 25–27 years) [40] at the first interview in which their BMI was obtained (Fig. 1). We used data from baseline interviews conducted from 2014 to 2015 and follow-up interviews conducted from 2018 to 2019. All interviews included in this study were conducted face-to-face using a standardised questionnaire that measured health, health-related behaviours and sociodemographic information [41].

### Outcome

Our outcome in this study was obesity measured as a binary variable. Participants over 18 years of age were considered to have obesity if their BMI was 30 kg/m^2^ or greater and participants under 18 years of age at the time of BMI measurement were classified in accordance with age-specific cut-offs [42, 43]. For participants with missing height and weight data from baseline interviews in 2014–2015, we used BMI data from the 2018–2019 follow-up interview to measure obesity. Twin pairs were excluded from all analyses if BMI data were missing at baseline and follow-up for both twins. If co-twin data was available for a twin with missing BMI data at baseline and follow-up, the twin who did not have missing was included in the twins-as-individuals analyses and not the within-between twin analyses (described subsequently).

### Ascertainment of exposure

Our primary exposure was high levels of internalising symptoms measured using the Strengths and Difficulties Questionnaire’s (SDQ) Internalising Scale [44] administered at baseline. The SDQ Internalising Scale is a 10-item self-report measure of socioemotional impact of internalising disorders in the last six months on a five-item sub-scale relating to internalising symptoms and a five-item sub-scale relating to the impact of internalising symptoms on social functioning. Participants rate items as occurring 0 “Not at all”, 1 “Somewhat” or 2 “Very Much” [45]. Mean scores from both sub-scales are multiplied by five to give a total score for each sub-scale [46]. Total SDQ score is calculated as the sum of the sub-scale totals for a maximum score of 20 [46]. Separate juvenile and adult versions of the SDQ were given to participants under and over the age of 18, respectively Adult and the standard juvenile versions of the SDQ have similar internal consistency and inter-scale correlations [47, 48]. We dichotomised the SDQ using a validated cut-off at the 90th percentile, those above and below this cut-off were considered to have high and low levels of internalising symptoms, respectively [46].

### Baseline measures

Self-reported covariates at baseline included smoking status (ever /never smoked), age of first alcohol consumption (≤14/>14 years old), and average daily recreational screen time (≤2/>2 h).

### Analysis

Descriptive statistics were calculated for all measures. We compared crude differences between high and low internalising groups at baseline using paired t-tests for continuous measures and χ^2^ tests for categorical measures overall, and by sex.

To estimate the association between internalising symptoms and obesity, we fitted two separate univariable and multivariable regression models: (i) a twins-as-individuals model and; and (ii) a within-between pair model. As sex has been found to be an effect modifier in previous studies [18], we fitted these regression models both for the total cohort and stratified by sex. The twins-as-individuals regression model simulated a typical cohort study analysis aimed at establishing individual risk factors. Both members from each twin pair are treated as unrelated individuals by adjusting for the paired nature of twin data and using a maximum likelihood approach to estimate the likelihood of obesity among those with high compared to low internalising symptoms [49].

To estimate the association between internalising symptoms and obesity while accounting for familial confounding (i.e., unmeasured genetic and environmental factors shared by twins), the ‘within-between’ pair model involved fitting a random-effects logistic regression model to estimate a ‘between-pair’ odds ratio and a ‘within-pair’ odds ratio. The ‘between-pair’ estimate represents the odds ratio using each twin pair’s mean level of high internalising symptoms (0 if neither twin has high internalising symptoms, 1 if one twin has high internalising symptoms and 2 if both twins in a pair have high internalising symptoms) to predict the odds of obesity. The ‘within-pair’ estimate represents the difference *within* twin pairs in the relationship between internalising symptoms and obesity [49]. The random effects estimator provides robust estimates of odds ratios when exposure data are missing from one twin in a twin pair [50].

Comparison of the between- and within-pair estimates provides evidence of the presence and strength of familial confounding by factors shared by twins [49]. For example, in the presence of a *between-pair* association, a *within-pair* association provides evidence that familial confounding partially explains the association between exposure and outcome. Conversely, in the presence of a *between-pair* estimate, not finding a *within-pair* association provides evidence that familial factors largely account for the association between high internalising symptoms and obesity [49].

All multivariable models were adjusted for smoking status, age of first alcohol consumption and recreational screentime. Smoking, alcohol consumption and physical activity are risk factors that are associated with internalising disorders and obesity [18]. As data on levels of physical activity were not collected, we used excess screentime as a proxy for physical activity as it is associated with physical activity, internalising disorders and obesity [51]. Excess screentime was self-reported average time using a computer or watching television outside of work and school. Individuals who reported a daily average greater than two hours were coded as experiencing excess screentime.

A likelihood ratio test was performed comparing model fit of the twins-as-individuals models to the within-between pair models for the whole sample, males only, and females only.

To test if sex modified the association between internalising symptoms and obesity, we used logistic regression models that included sex as a covariate and an interaction term for sex and internalising symptoms. This interaction was examined using likelihood ratio tests to compare model fit with and without an interaction term. For the ‘within-between’ pair regression analysis, the sex and internalising symptoms interaction term was only included in the *between-pair* and not the *within-pair* model covariate as all twins in our cohort were from same-sex twin pairs.

To assess if dichotomising SDQ internalising symptoms score and BMI may have resulted in a loss of statistical power, we performed supplementary twins-as-individuals and within-between pair linear regression analyses. We used the sample standard deviation of SDQ internalising score and BMI as exposure and outcome, respectively.

We used chi-squared tests to determine if there were differences in the proportion of missing BMI data between those with high and low internalising symptoms. To assess if our results may have been impacted from using measurement of BMI from follow-up for those who had a missing BMI value at baseline, we conducted sensitivity analyses whereby we restricted analysis to participants with baseline BMI data (Supplementary Table 1).

We also assessed the impact of socioeconomic position (SEP) on missing BMI data at the twin pair-level. Pairs with missing BMI data from one or both twins were compared to pairs with no missing BMI data. Socioeconomic position was operationalised using the highest level of parent education scaled using the International Scale of Education, in line with previous studies using the Twin Life cohort [52]. As missing data and SEP were evaluated at the twin-pair level, missing data and SEP variable were the same within twin-pairs, and only one twin from each twin-pair were included in regression analysis.

All data analysis was conducted using Stata Version 15.1 [53].

## Results

From a total cohort of 2044 twin pairs (4088 individual twins), we excluded 2 twin pairs with unknown zygosity, 10 pairs where one or both twins were missing SDQ data, and 23 pairs where both twins were missing BMI data from baseline and follow-up. The remaining 2009 twin pairs (4018 individuals) were included in the within-between pair analyses. A further 212 individual twins with missing BMI data were excluded from the twins-as-individuals analysis, but included in the within-between pair analyses. Of the 4018 twin individuals included in analyses, 3614 individuals had BMI data available from baseline measurement in 2014 or 2015 and 192 individuals had BMI data from follow-up only in 2018 or 2019. The proportion of BMI data missing did not differ for individuals with high and individuals with low internalising symptoms (*p* = 0.278), but there were 0.90 times lower odds of missing BMI at wave 3 for each one unit increase in parental education (OR 0.90, 95% CI 0.87, 0.93).

An overview of the participant characteristics by internalising symptoms status is presented in Table 1. Participants were aged 16 to 25 years at baseline (mean = 19.9 years, SD 3.1) and there were more females (*n* = 2314, 57.6%) than male participants (*n* = 1704, 42.4%). A total of 4.2% participants were obese at their first recorded BMI, 13.1% of participants reported high internalising symptoms and 86.9% reported low internalising symptoms.

**Table 1:** Summary statistics stratified by sex and internalising symptom severity.

	Whole sample (*n* = 4018)			Female (*n* = 2314)			Male (*n* = 1704)		
	High internalising (*n* = 527)	Low Internalising (*n* = 3491)	p-value*	High internalising (*n* = 385)	Low Internalising (*n* = 1929)	p-value*	High internalising (*n* = 142)	Low Internalising (*n* = 1562)	p-value*
Age at baseline in years mean (SD)	20.9 (2.9)	19.8 (3.1)	<0.001	20.8 (3.0)	19.8 (3.1)	<0.001	21.4 (2.7)	19.7 (3.1)	<0.001
BMI mean (SD)	22.5 (4.2)	22.2 (3.6)	0.081	22.2 (4.3)	21.6 (3.5)	0.003	23.2 (3.9)	22.9 (3.6)	0.355
Obese at first measurement of BMI	7.1 (35)	3.7 (124)	0.001	6.6 (24)	3.0 (54)	0.001	8.3 (11)	4.7 (70)	0.066
Ever smoker	36.1 (189)	28.0 (967)	<0.001	33.6 (129)	23.3 (445)	<0.001	42.9 (60)	33.8 (522)	0.031
Drank alcohol before age 14	19.7 (104)	18.5 (644)	0.479	20.8 (80)	19.5 (377)	0.309	16.9 (24)	17.1 (267)	0.954
Greater than 2 h of screentime/day	90.3 (374)	91.2 (2679)	0.572	89.3 (276)	89.6 (1465)	0.905	93.3 (98)	93.2 (1214)	0.971

*two tailed *p*-values. Chi square tests were used to test for group differences for categorical variables and two sampled *t*-tests for continuous outcomes

Participants with high internalising symptoms were a year older on average, (*t* = −8.2, *p* < 0.001), more likely to be female than male (χ^2^ = 59.4, *p* < 0.001), and more likely to be ever smokers than never-smokers (χ^2^ = 14.5, *p* < 0.001) when compared to participants with low internalising symptoms. Sex-stratification indicated BMI was higher in females with high internalising symptoms than low internalising symptoms (mean difference = 0.6 kg/m^2^, *p* = 0.003), but there was no difference in mean BMI for males with high or low internalising symptoms (mean difference = 0.3 kg/m^2^, *p* = 0.355). There was no evidence of differences between participants with high internalising or low internalising in any other covariates measured. Smoking was more prevalent in both females (χ^2^ = 18.3, *p* < 0.001) and males (χ^2^ = 4.7, *p* < 0.031) with high internalising symptoms compared to those with low internalising symptoms. There were no other significant sex-specific differences in baseline variables.

### Twins-as-individuals analyses

After adjusting for covariates, our twins-as-individuals analysis showed that high internalising symptoms were associated with a 3.1 (95% CI: 1.5, 6.8) times higher odds of obesity when compared to individuals with low internalising symptoms (Fig. 1). Sex-stratified twins-as-individuals analyses found that, after adjusting for covariates, in females there was a 4.1 (95% CI: 1.5, 11.1) times higher odds of obesity in those with high compared to low internalising symptoms. However, for males there was no association between internalising symptoms and obesity (Table 2). There was little evidence that sex modified the association between internalising symptoms and obesity (likelihood ratio test *p* = 0.051).

**Table 2:** Estimates from twins-as-individuals and between within-pair analyses.

		Odds of obesity				Odds of obesity			
		Unadjusted OR (95% CI)	*p*-value	*N*	Likelihood ratio test (*p*-value)	Adjusted OR (95% CI)*	*p*-value	*N*	Likelihood ratio test (*p*-value)
Whole sample									
High compared to low levels of internalising symptoms	Twins as individuals	2.7 (1.4, 5.2)	0.002	3806^a^		3.1 (1.5, 6.8)	0.003	3173^a^	
	‘Between-pair’	4.2 (1.5, 11.7)	0.007	2009^b^		6.2 (1.7, 22.8)	0.006	1862^b^	
	‘Within-pair’	2.0 (0.9, 4.7)	0.112	2009^b^	0.292	1.9 (0.7, 5.2)	0.202	1862^b^	0.448
Females									
High compared to low levels of internalising symptoms	Twins as individuals	3.2 (1.4, 7.6)	0.007	2185^a^		4.1 (1.5, 11.1)	0.005	1837^a^	
	‘Between-pair’	7.9 (1.8, 33.5)	0.005	1157^b^		12.9 (2.2, 76.8)	0.005	1074^b^	
	‘Within-pair’	1.7 (0.6, 5.1)	0.340	1157^b^	0.091	1.8 (0.5, 6.5)	0.353	1074^b^	0.346
Males									
High compared to low levels of internalising symptoms	Twins as individuals	2.7 (1.0, 7.8)	0.061	1621^a^		2.8 (0.8, 10.0)	0.118	1336^a^	
	‘Between-pair’	2.7 (0.5, 16.5)	0.270	852^b^		5.1 (0.6, 47.1)	0.151	788^b^	
	‘Within-pair’	2.7 (0.7, 11.2)	0.165	852^b^	0.995	2.0 (0.4, 10.2)	0.403	788^b^	0.498

The reference category for all odds ratios is participants with low internalising symptoms.

Likelihood ratio tests compared model fit for the twins as individuals model to the between-within-pair model to determine if addition of the within-pair estimator imporoved model fit a indicates individual twins. ^a^indicates number of individual twins ^b^indicates number of twin pairs.

*Adjusted for ever smoking, age at first alcohol consumption, and daily recreational screentime.

### Within-between pairs analyses

**Fig. 2:**
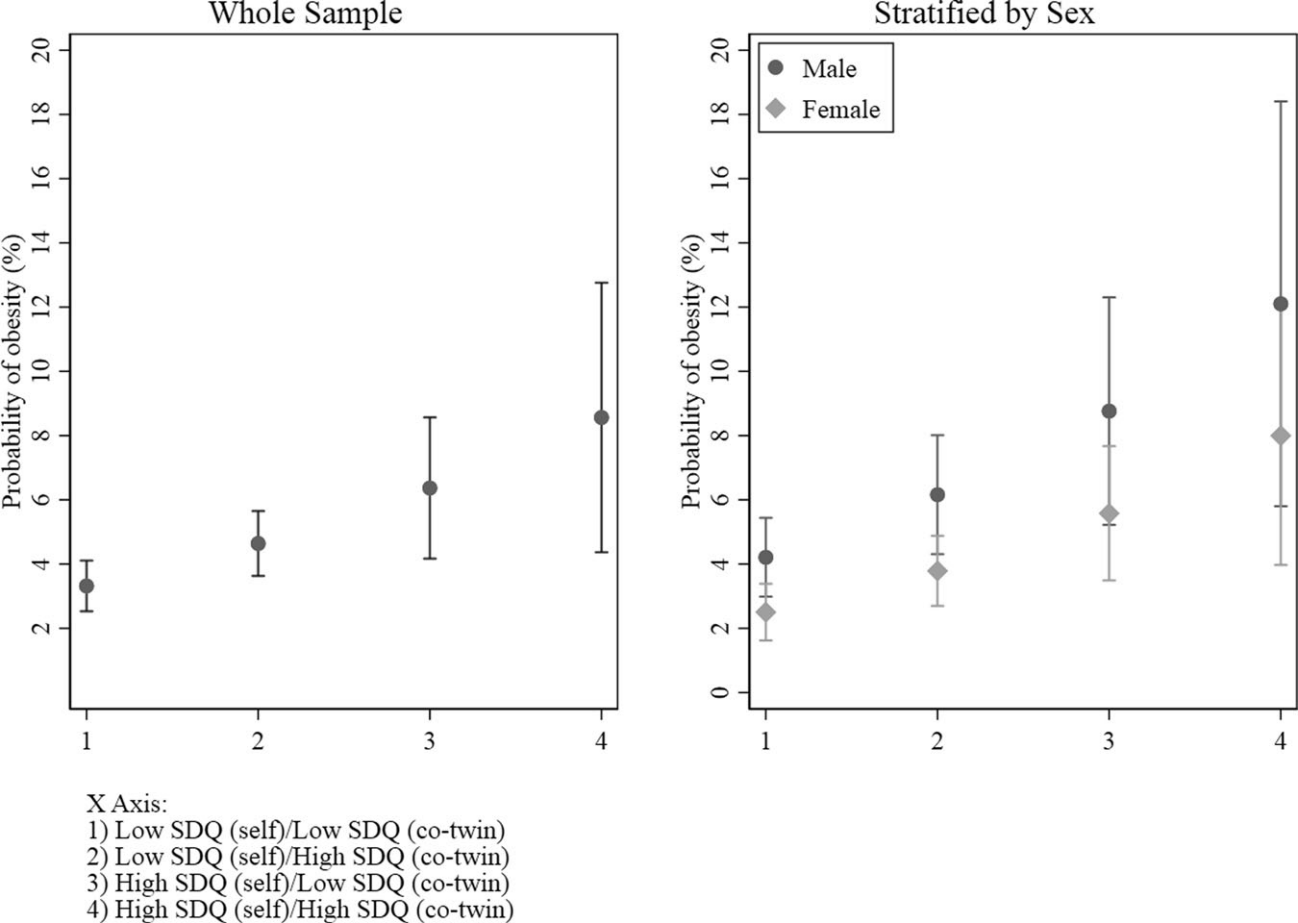
Between-pair odds of obesity indicating probability of obesity for individuals by their own internalising symptom level and the internalising symptom level of their co-twin. The graph on the left depicts the whole sample and the graph on the right depicts analyses stratified by sex. Error bars indicate 95% confidence intervals.

Our between-pair analyses found the adjusted odds of obesity were 6.2 (95% CI: 1.7, 22.8) times higher in twin pairs with high internalising symptoms compared to twin pairs with low internalising symptoms (Fig. 2). However, our adjusted within-pair analyses found no evidence of an association between internalising symptoms and obesity after adjusting for familial confounding (Table 2).

When we stratified our between-pair analyses by sex, after adjusting for covariates, there was a 12.9 (95% CI: 2.2, 76.8) times higher odds of obesity in female pairs with high compared to low internalising symptoms (Table 2). However, no significant differences were observed in the within-pair odds of obesity in females with high compared to low internalising symptoms (Table 2). No between- or within-pair associations between internalising symptoms and obesity were observed for male twin pairs in the adjusted analyses (Table 2). In analyses that included the whole sample and analyses that stratified by sex, the probability of obesity increased if an individual’s co-twin had high internalising symptoms, even if they did not have high internalising symptoms themselves. The estimated probability of obesity was greatest for individuals who had high internalising symptoms and who had a co-twin with high internalising symptoms (Fig. 2).

A likelihood ratio test comparing regression models that did and did not allow between-pair internalising symptoms to vary by sex found weak evidence of effects modification by sex (*p* = 0.037).

Likelihood ratio tests found no difference in model fit when comparing the twins-as-individuals models to the within-between pair models for unadjusted and adjusted models that featured the whole sample or models that analysed males and females separately (Table 2).

Supplementary analyses using standard deviation of SDQ internalising score (3.5 points) and BMI (3.7 kg/m^2^) found associations between internalising symptoms and obesity attenuated to the null after adjusting for within-pair familial factors shared by twins (supplementary Table 2). This pattern was observed for the whole sample, females, and males.

Sensitivity analyses using only BMI data collected from baseline interviews did not indicate any associations between internalising disorders and obesity.

## Discussion

Our study investigated the extent to which familial confounding affected the association between internalising symptoms and obesity in adolescent and young adult twins. In analyses that utilised members of a twin pair as individuals (mimicking a typical cohort study), we found that high levels, compared to low levels, of internalising symptoms were associated with an increased odds of obesity among adolescents and young adults. Sex-stratified analyses indicated that high internalising symptoms increased the odds of obesity in females, but there was little evidence of an association between internalising symptoms and obesity in males.

Between- and within-twin pair analyses found that, for the whole sample, accounting for familial factors led to a 60% attenuation in the within-pair association between high internalising symptoms and obesity reduced (Table 2). However, as likelihood ratio tests indicated that accounting for within-pair (i.e. shared genetic and environmental factors) effects did not improve model fit, we have little evidence against the null hypothesis that an individual twin’s risk of obesity is only dependent on their level of internalising symptoms relative to their co-twin’s level of internalising symptoms. These results do not provide formal evidence of familial confounding, but indicate that familial factors shared by twins and individual factors not shared by twins are both likely to contribute to the association between internalising symptoms and obesity.

Sex-stratified analyses found that the within-pair association between internalising and obesity in females attenuated to the null after adjusting for within-pair factors, but no formal evidence of familial confounding. We found no associations between internalising symptoms and obesity in males.

Our findings that familial factors partially confound the association between high internalising symptoms and obesity among adolescent and young adult females are congruent with previous co-twin studies in adult samples that found that familial factors confounded the association between symptoms of depression or anxiety and obesity in adult samples [35, 37]. When taken together, our study and previous research provide evidence of familial confounding in the association between internalising disorders and obesity across adolescence, young adulthood and in middle-aged adults [35, 37]. Therefore, typical cohort studies that have found associations of internalising symptoms and obesity without accounting for family factors may be subject to bias and have likely overestimated the causal effect of internalising disorders on obesity.

This indicates efforts to prevent and treat obesity should target the siblings of individuals with internalising disorders where possible, as they are at elevated risk of obesity.

We found evidence that in females both the individual twin and her co-twin’s levels of internalising symptoms affect that individual’s risk of obesity. We found no evidence of an association between internalising disorders and obesity in males, which is broadly consistent with previous research [37]. Studies of unrelated individuals have found a stronger association between internalising disorders and obesity in females compared to males [9, 10], however our findings raise the possibility that family factors may impact internalising symptoms and obesity to a greater extent among young females than young males.

Our study was likely limited by a lack of statistical power. Less than 4% of our sample were obese when the first measurement was taken, a lower prevalence than the estimated 10% prevalence of adolescent obesity in the general German population [54]. A lack of statistical power could explain why, despite there being a strong attenuation of the within-pair effects after accounting for familial confounding in the within-between pair model, likelihood ratio tests indicated no formal evidence of familial confounding. This was likely impacted by our use of dichotomous measures of internalising symptoms and obesity. Supplementary analyses using found associations between continuous measures of internalising symptoms and obesity were associated, but attenuated to the null after accounting for familial factors shared by twins. This pattern was observed for the whole sample, males, and females.

A limitation of the co-twin study design we utilised is that it accounts for the combined effect of shared genetic variants and environmental risks for internalising symptoms and obesity. As a result, our findings do not provide evidence of the extent to which specific genetic or environmental risks shared by twins confound the association between internalising disorders and obesity. Studies that employed classic twin and family designs to investigate the contributions of shared genetic variance and environmental risks have produced conflicting findings. Two studies found the shared genetic variance for internalising disorders and obesity of 12 and 15% with no contribution from shared environmental factors [24, 25]. However, two other studies found no shared genetic variance and attributed all familial confounding to shared environmental factors [26, 27]. However, there is evidence from genome wide association, Mendelian randomisation, and linkage disequilibrium regression studies that there are shared genetic risks for depression and body mass [55, 56].

Further research is required to investigate how specific environmental factors interact with genetic vulnerabilities shared by families and apply this understanding to identify family determinants of health that are promising targets for preventative and clinical interventions.

Our use of a cross-sectional design meant we cannot provide evidence of the temporal onset of internalising disorders, obesity, and the effects of familial confounding. Consequently, our findings should only be interpreted in relation to the cross-sectional association between high internalising symptoms and obesity without any inference as to the temporal sequence of morbidity. However, a previous Mendelian randomisation study of an adult sample (age range 37–73) found evidence that BMI was a causal risk for depression, but no evidence that depression was a causal risk for BMI [57]. Further longitudinal research should build on these findings and clarify the causal direction of this association in adolescence and young adulthood.

Our findings of decreasing attrition being inversely related to socioeconomic position mean our results may not be generalisable to adolescents who experience lower familial socioeconomic position. Furthermore, our study was conducted prior to the 2020 COVID-19 pandemic, a period associated with increased prevalence of internalising disorders [58]. Thus, our findings may not be generalisable to peri- and post- COVID-19 pandemic periods.

## Conclusions

Environmental and genetic variance shared by twins partially confounded the association between internalising disorders and obesity from adolescence and across adulthood. As a result, clinical treatment and prevention of internalising disorders and obesity should incorporate the familial environment in conjunction with a focus on treating and preventing internalising disorders to reduce the burden of obesity. Additionally, family members of those with internalising disorders should be incorporated into treatment and prevention efforts as they are at increased risk of obesity. This is particularly important in females who are at increased risk of co-occurring high levels of internalising symptoms and obesity than males.

### Supplementary information

Supplementary Table 1

Supplementary Table 2

## Data Availability

The .do file used in our analysis is available from https://github.com/Alex-Campbell01/Internalising_Obesity_Co-Twin/blob/main/Cotwin_internalising_obesity_final.do. This study received approval by the University of Melbourne Human Research Ethics Council (reference: 2021-21305-16046-2) and informed consent was obtained from all participants. Data are available to researchers free of charge upon request in the GESIS database catalogue as a Scientific Use File. For details see: (https://www.twin-life.de/documentation/overview-and-getting-started/data-access).
